# Adaptive selection of founder segments and epistatic control of plant height in the MAGIC winter wheat population WM-800

**DOI:** 10.1186/s12864-018-4915-3

**Published:** 2018-07-31

**Authors:** Wiebke Sannemann, Antonia Lisker, Andreas Maurer, Jens Léon, Ebrahim Kazman, Hilmar Cöster, Josef Holzapfel, Hubert Kempf, Viktor Korzun, Erhard Ebmeyer, Klaus Pillen

**Affiliations:** 1 Chair of Plant Breeding, Martin Luther University Halle-Wittenberg, Betty-Heimann Straße 3, 06120 Halle, Germany; 2 Institute of Crop Science and Resource Conservation, Crop Genetics and Biotechnology Unit, University of Bonn, Katzenburgweg 5, Bonn, Germany; 3 Syngenta Seeds GmbH, Kroppenstedter Straße 4, 39387 Oschersleben (Bode), Hadmersleben, Germany; 4 RAGT 2n, Steinesche 5A, 38855 - Silstedt, Wernigerode, Germany; 5 Secobra Saatzucht GmbH, Feldkirchen 3, 85368 Moosburg an der Isar, Germany; 6 KWS SAAT SE, Grimsehlstraße 31, 37555 Einbeck, Germany; 7 KWS LOCHOW GMBH, Ferdinand-Lochow-Straße 5, 29303 Bergen/Wohlde, Germany

**Keywords:** Winter wheat, Multi-parent advanced generation intercross (MAGIC), Plant height, Genome-wide association study (GWAS), Quantitative trait loci (QTL), Segregation distortion, Epistatic effects, Single nucleotide polymorphism (SNP)

## Abstract

**Background:**

Multi-parent advanced generation intercross (MAGIC) populations are a newly established tool to dissect quantitative traits. We developed the high resolution MAGIC wheat population WM-800, consisting of 910 F_4:6_ lines derived from intercrossing eight recently released European winter wheat cultivars.

**Results:**

Genotyping WM-800 with 7849 SNPs revealed a low mean genetic similarity of 59.7% between MAGIC lines. WM-800 harbours distinct genomic regions exposed to segregation distortion. These are mainly located on chromosomes 2 to 6 of the wheat B genome where founder specific DNA segments were positively or negatively selected. This suggests adaptive selection of individual founder alleles during population development. The application of a genome-wide association study identified 14 quantitative trait loci (QTL) controlling plant height in WM-800, including the known semi-dwarf genes *Rht-B1* and *Rht-D1* and a potentially novel QTL on chromosome 5A. Additionally, epistatic effects controlled plant height. For example, two loci on chromosomes 2B and 7B gave rise to an additive epistatic effect of 13.7 cm.

**Conclusion:**

The present study demonstrates that plant height in the MAGIC-WHEAT population WM-800 is mainly determined by large-effect QTL and di-genic epistatic interactions. As a proof of concept, our study confirms that WM-800 is a valuable tool to dissect the genetic architecture of important agronomic traits.

**Electronic supplementary material:**

The online version of this article (10.1186/s12864-018-4915-3) contains supplementary material, which is available to authorized users.

## Background

Bread wheat (*Triticum aestivum* L*.,* 2n = 6× = 42, AABBDD) is one of the three most important crop species globally, with a world production of more than 710 million metric tons [1]. High-resolution QTL studies are still challenging in wheat due to the large genome size (17 GB), polyploid nature and huge amount of repetitive DNA present in wheat. Today, a high genetic marker density is no longer a limiting factor for QTL studies in wheat through the recent advances in genotyping techniques [2]. Therefore, there is an increasing demand for large and complex mapping populations. This enables to study the effects of allelic diversity at a higher genetic resolution and statistical power. Bi-parental populations, used for decades in QTL mapping, estimate allelic effects with high statistical power but suffer from a low genetic diversity regarding the worldwide allelic diversity present in wheat [1]. In contrast, association panels try to capture a high amount of genetic diversity by selecting defined samples from the whole population. This leads to high genetic diversity within the panel but reduced statistical power to detect rare allele effects [1]. Therefore, multi-parent populations are proposed to build a bridge between the two classical QTL mapping populations. They combine higher genetic diversity among the offspring based on an increased number of founders with higher statistical power [1]. The most noteworthy designs, which were successfully used to study multi-parental populations, are nested association mapping (NAM) [3, 4] and multi-parent advanced generation intercrosses (MAGIC), the latter following a mating design according to Cavanagh et al. [5]. MAGIC populations proved their usefulness in several species like *Arabidopsis* [6], rice [7], barley [8], maize [9], tomato [10] and wheat [2, 11–13]. So far, two bread wheat MAGIC populations are established. The first was developed by inter-mating four Australian spring wheat cultivars pioneering the setup and statistical analysis of MAGIC populations in crops [11]. The NIAB MAGIC population was second, developed by intercrossing eight winter wheat founders selected to represent the diversity of the UK wheat germplasm [2]. Its usefulness was proved by locating genes controlling the presence of awns [2]. Subsequently, Gardner et al. [13] constructed a population specific genetic map for the MAGIC NIAB population using R package mpMap [14]. The same statistical methods were applied in a four-way durum wheat MAGIC population as a proof of concept [12]. The NIAB-MAGIC population was used to map genes regulating plant senescence in wheat [15].

Plant height is a key trait in wheat as it affects grain yield, grain quality [16] and pathogen resistance, for instance against *Fusarium* [17]. The introduction of several *reduced height (Rht)* genes controlling the gibberellic acid (GA) pathway in wheat, served as a catalyst for the green revolution [18]. The semi-dwarf alleles of *Rht-B1* on 4B and *Rht-D1* on 4D represented major sources to achieve reduced plant height in European wheat breeding programs since the 1960s. This resulted in markedly increased grain yields in most environments [19]. The semi-dwarfing gene *Rht8* is widely used in dry environments of Southern and Eastern European environments [20]. Further, several other semi-dwarfing genes are known, like *Rht4, Rht5, Rht6* [21], *Rht7* [22]*, Rht9* [23], *Rht11* [24], *Rht12* [25], *Rht13* to *Rht20* [24], however, they are not yet integrated in European wheat breeding programs.

In addition to the known *Rht* genes, several genetic regions were identified by QTL analysis investigating plant height in various wheat mapping populations [11, 16, 26, 27]. A number of plant height QTL could be mapped in each population. However, all populations shared the semi-dwarf effects of the major plant height genes *Rht-B1* and *Rht-D1*, supporting the idea that these two genes are widely used in modern wheat breeding to control plant height.

Epistasis, the genetic interaction between independent loci, is one additional factor known to play an important role in dissecting quantitative traits [28]. Epistasis has been investigated in crops like maize [29], potato [30], barley [4, 31] and wheat [16, 32, 33]. The relevance of epistasis has further been proven through genomic selection studies. An increase in prediction accuracy was reported when modelling main and epistatic effects [34, 35]. The authors stated that a large population size and a high marker density are necessary pre-requisites to apply a full marker by marker model and to successfully estimate the contribution of epistasis to the regulation of quantitative traits.

In this study, we report on the new MAGIC winter wheat population WM-800. WM-800 was characterized based on genotypic data derived from a 15 k Infinium iSelect SNP array. This data was applied to investigate genetic diversity, linkage disequilibrium, allele frequency and segregation distortion. We further proved that WM-800 is a powerful mapping tool by dissecting the genetic architecture of plant height. We also demonstrated that epistatic interactions play a significant role controlling plant height in WM-800.

## Methods

### Development of WM-800

WM-800 was developed following the crossing scheme reported by Cavanagh et al. [5]. The WM-800 founders are eight European winter wheat cultivars (Table 1, Additional file 1: Table S1) released between 2008 and 2017. They were selected based on their market dominance regarding yield, baking quality and pathogen resistance. Together, the founders represented a market share of 14,582 ha (31%) of the German certified seed production in 2014 (Table 1). They were crossed by RAGT 2n and Syngenta in four pairs to create the F_1_ seeds: Patras x Meister (AB), Linus x JB Asano (CD), Tobak x Bernstein (EF) and Safari x Julius (GH). Single F_1_ plants of each 2-way cross were intercrossed, resulting in two 4-way crosses (ABCD) and (EFGH). Subsequently, 141 seeds of the 8-way cross (ABCDEFGH) were produced by Syngenta, where 56 seeds derived from intercrossing 18 4-way F_1_ plants (ABCD x EFGH) and 85 reciprocal 4-way F_1_ plants (EFGH x ABCD), respectively, to assure a balanced allele frequency for each of the eight founders. On average 15 selfed F_2_ seeds per 8-way plant were produced. These seeds were further advanced by single seed descent (SSD) to yield 2125 F_4_ recombinant inbred lines (RILs). Each WM line is expected to hold 12.5% of the genome of each of the eight WM founders. Altogether, 1323 WM lines were randomly selected and analysed with KASP marker on the presence of double dwarfism at KWS SAAT SE, Einbeck. Out of these, 935 RILs were not homozygous at both loci for the dwarfing allele *Rht-B1* and *Rht-D1* according to the KASP marker analysis. These were propagated (F_4:5_) for one round of bulk propagation to establish the MAGIC wheat population WM-800, which is composed of a final set of 910 RILs in F_4:6_.

**Table 1:** Founder information and plant height descriptive statistics for WM-800 lines and founders

Genotype^a^	Breeder	Release	Multiplication area 2014 (ha)	Quality group	Crossing position^b^	Rht-B1^c^	Rht-D1^d^	N^e^	HEI^f^	SD^g^	Min^h^	Max^i^	CV (in %)^j^	h^2k^
Patras	DSV	2012	2884	A	A	Rht-B1a	**Rht-D1b**	7	82.0	4.0	77.0	87.0	4.83	
Meister	RAGT	2010	212	A	B	Rht-B1a	Rht-D1a	7	81.1	7.2	75.0	95.0	8.93	
Linus	RAGT	2010	917	A	C	Rht-B1a	**Rht-D1b**	7	78.3	4.0	74.0	84.0	5.09	
JB Asano	Breun	2008	4044	A	D	Rht-B1a	**Rht-D1b**	22	83.4	4.9	71.0	92.0	5.88	
Tobak	W von B	2011	3385	B	E	**Rht-B1b**	Rht-D1a	60	83.8	4.6	69.0	93.0	5.46	
Bernstein	Syngenta	2014	154	E	F	Rht-B1a	Rht-D1a	10	96.0	9.2	82.0	107.0	9.58	
Safari	Syngenta	2017		C	G	**Rht-B1b**	Rht-D1a	7	81.9	4.1	75.0	87.0	5.06	
Julius	KWS	2008	2986	A	H	Rht-B1a	**Rht-D1b**	8	86.9	5.5	76	92	6.29	
Founder total or mean			14,582					128	84.2	6.4	69.0	107.0	7.55	0.90
WM-800 total or mean								910	80.1	11.2	45.5	112.0	14.30	0.90

^a^Discrimination Founders and MAGIC-WHEAT WM-800 lines

^b^Position in crossing scheme of the WM-800

^c^*Rht-B1* genotype based on SNP TG0010a (bold genotype causes semi-dwarfism)

^d^*Rht-D1* genotype based on SNP TG0011a (bold genotype causes semi-dwarfism)

^e^Number of observations

^f^Plant height Lsmeans (in cm)

^g^Standard deviation

^h^Minimum

^i^Maximum

^j^Coefficient of variation [in %]

^k^Heritability

### Measuring plant height in WM-800

Bulk propagation of WM-800 RILs took place at Syngenta’s breeding station in Hadmersleben, Germany (51°98′29.07′´ N; 11°29′93.28″ E). Plant height (HEI) was measured in one replication on single plants in 2015 and in one replication in field plots (1.50 × 2.20 m with 330 viable seeds/m^2^) in 2016. Subsequently, analysis of variance for plant height was carried out with SAS 9.4 (SAS Institute Inc., Cary, NC, USA) using *PROC MIXED* to test for genotype and year effects. Least squares means (LSmeans) per genotype were calculated using *PROC MIXED* (SAS 9.4) assuming fixed genotype effects and random year effects. *PROC VARCOMP* (SAS 9.4) was used to estimate variance components. Broad-sense heritability (h^2^) was calculated applying the formula h^2^ = V_G_ / (V_G_ + V_GY_/y + V_R_/(y*r)), where V_G_, V_GY_ and V_R_ denote the variance components genotype, genotype by year and residual, respectively. Y and r denote the number of years and replicates per genotype, respectively.

### Genotyping single nucleotide polymorphism (SNP) markers in WM-800

Bulked DNA from 12 F_4:5_ seedlings was extracted for 935 MAGIC wheat lines at TraitGenetics, Gatersleben, Germany (www.traitgenetics.com). Infinium 15 k iSelect SNP array was used for genotyping. It contains 13,006 SNPs selected from the wheat 90 k SNP array [36], including a small set of known genes like *Ppd, Rht, Vrn-1 and Vrn-2*.

Polymorphic SNP data was quality checked. SNPs with missing data > 10%, heterozygous genotypes > 10% and minor allele frequency < 1% were removed [37]. The genetic positions published in the wheat consensus map [36] were used as a reference to assign SNPs to wheat chromosomes and place them in a linear order. SNPs, which could not be assigned to the wheat consensus map [36] were fitted using chi-square test [4]. Genotype data was transcribed into a binary code based on an identity-by-state (IBS) matrix according to the presence of the Julius founder allele. For this, homozygous genotypes carrying two Julius (J) or Non-Julius alleles (N) were assigned a value of 2 and 0, respectively. Heterozygous genotypes were assigned a value of 1. Missing genotypes were predicted applying the mean imputation (MNI) approach [38]. Each missing SNP value was replaced by the mean SNP value calculated across all WM-800 genotypes. We selected Julius as the reference founder since the cultivar was selected for the 10 Wheat Genomes Project (see: www.wheatinitiative.org) and since a BLAST database of wheat genome assemblies, including Julius, is available (see webblast.ipk-gatersleben.de/wheat_ten_genomes/).

### Allele frequency and segregation distortion in WM-800

The IBS-SNP matrix was used to calculate allele frequencies and segregation distortion (SD) for each SNP in WM-800. Bi-allelic SNPs do not follow a Mendelian segregation in an 8-way MAGIC population. The segregation ratio of the two alternative SNP alleles in the population depends on their frequency in the founder set. Thus, the SNPs were classified into seven allele frequency groups (AFG 1 – AFG 7), depending on the presence of the Julius allele among the founders. Segregation distortion within each AFG was estimated by chi-square testing, using *PROC FREQ* (SAS 9.4). Subsequently, Bonferroni-Holm correction of the *p*-values (p_Bon-Holm_) was used for multiple testing with *PROC MULTTEST* (SAS 9.4) [39]. A chromosomal segregation distortion region (SDR) was defined if at least three adjacent SNP loci showed a significant segregation distortion with p_Bon-Holm_ < 0.01 [40]. AFG 1 and AFG 7 are of particular interest, because allele effects can be assigned to a single founder within these AFGs. The allele effect can be directly assigned to founder Julius in AFG 1 with a founder allele segregation of 1 (Julius allele) to 7 (Non-Julius allele). In addition, AFG 7 with a founder allele segregation of 7 (Julius allele) to 1 (Non-Julius allele) can be further divided into seven subgroups according to the WM-800 founder carrying the Non-Julius allele.

### Genetic similarity and linkage disequilibrium in WM-800

Procedure *PROC DISTANCE* (SAS 9.4) with method simple matching was used to calculate genetic similarity (GS) between WM-800 lines and their eight founders. Subsequently, population structure within WM-800 and their founders was investigated based on GS values applying a principal component analysis (PCA) with *PROC PRINCOMP* (SAS 9.4).

Linkage disequilibrium (LD) was calculated as the squared allelic correlation linkage (r^2^) [41] using the R packages “genetics” and “LDHeatmap”. The estimated r^2^ values were plotted against the genetic distance according to [36]. The second-degree smooth locally weighed polynomial regression (LOESS) curve was estimated with *PROC LOESS* (SAS 9.4). A population-specific background LD (r^2^) was estimated as the 95th-percentile of r^2^ values for unlinked markers [42].

### Genotypic data and genome-wide association study (GWAS)

The multiple linear regression model “Model-A” was used to conduct GWAS mapping based on plant height LSmeans for each WM-800 line. The model [43] was successfully adapted to a barley MAGIC population [44]. Analysis was carried out with *PROC GLMSELECT* (SAS 9.4) including 20 times five-fold cross-validation. Stepwise forward and backward selection of significant SNPs was conducted with model selection criteria of *p* ≤ 0.001. SNPs were accepted as significant marker-trait associations (MTA) if they were included in the final model in at least 35 cross-validation runs (detection rate ≥ 35%) and if p_Bon-Holm_ < 0.01 after adjusting *p*-values for multiple testing with the Bonferroni-Holm procedure using *PROC MULTTEST* (SAS 9.4) [39]. MTAs were grouped to a QTL if significant SNPs were placed in a window of ≤5 cM and if they revealed additive effects of the same direction [4].

Epistatic effects between SNPs were estimated with the *PROC MIXED* (SAS 9.4) procedure including a five-fold cross validation based on LSmeans of WM-800 lines as input and a marker 1 by marker 2 interaction to test for fixed interaction effects including a forward selection procedure. At first, markers with a single marker probability below 0.15 were included in pairwise marker interaction analysis. Second, a forward and backward selection procedure was applied for all significant marker by marker combinations. A FDR corrected p-value of ≤0.01 was chosen as a significance threshold to accept the presence of an epistatic effect. Explained marker by marker variance was calculated by comparing the sum of squares of the respective M by M interaction to the sum of squares of the genotypes. Values are given in percent. Marker interactions were grouped to a single interaction if significant SNP interactions were placed in a window ≤7 cM. Subsequently, the additive by additive epistatic effect (aa) was estimated [45]: aa = (JJ + NN) – (JN + NJ) where JJ and NN represent the mean performances of WM-800 lines possessing two Julius, respectively two Non-Julius genotypes, at the two interacting SNPs. JN and NJ represent the mean performances of WM-800 lines possessing a Julius genotype at the first SNP and a Non-Julius genotype at the second SNP and vice versa.

## Results and discussion

### Phenotypic characterization of WM-800

**Fig. 1:**
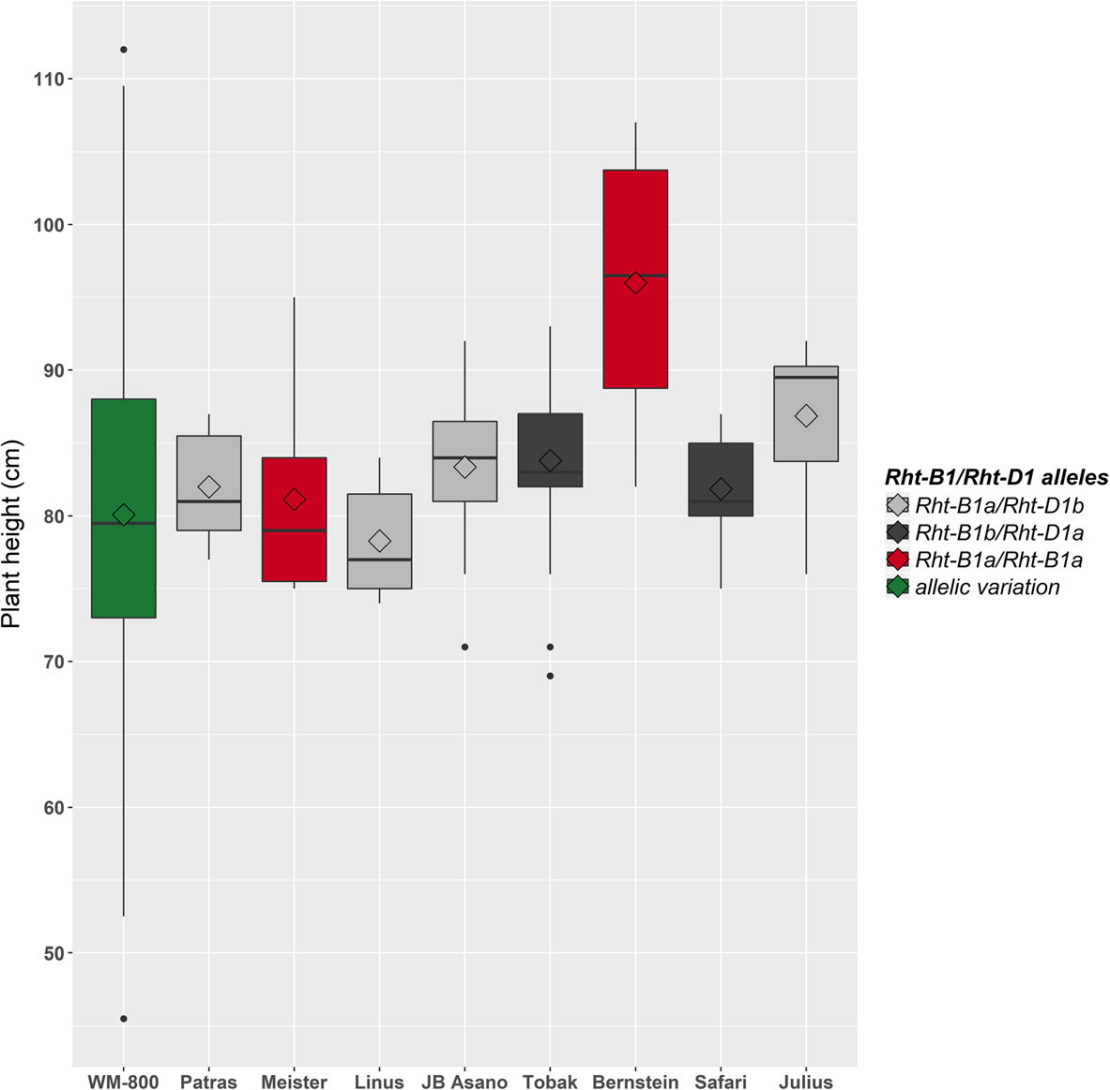
Plant height variation among WM-800 lines and founders in regard to *Rht-B1* and *Rht-D1* alleles

For plant height, a high level of diversity was observed in population WM-800 displaying a range from 45.5 to 112.0 cm (Fig. 1, Table 1). Detailed information on plant height variation in WM-800 and founder cultivars is given in Table 1 and in Additional file 1: Table S2. On average, WM lines were shorter than the founders with Lsmeans of 80.1 and 84.2 cm, respectively (Table 1). In contrast, WM lines displayed a higher coefficient of variation than the founders with 14.25 and 7.55%, respectively. This observation indicates the presence of transgressive segregation in WM-800, where 42 and 79 WM lines are significantly taller and shorter than the tallest (Bernstein) and shortest (Linus) founder cultivar, respectively (Additional file 1: Table S2). In addition, broad sense heritability among WM lines was high with h^2^ = 0.90 (Table 1). Both findings, transgressive segregation and a high level of heritability, are supportive to locate loci controlling plant height in WM-800 based on a genome-wide association study.

### SNP genotyping in WM-800

Altogether, 7849 polymorphic SNPs were genotyped in WM-800 (Additional file 1: Table S1 and S3). Out of these, 6721 SNPs were assigned to a genetic position [36]. In addition, 863 SNPs (11%) were mapped based on chi-square association with mapped SNPs [4]. The residual 265 SNPs remained unassigned. About 41.0, 48.2, and 10.7% of the SNPs were mapped to the A, B, and D subgenomes, respectively. The map length amounted to 3588 cM, with individual chromosome lengths ranging from 114.9 cM (4B) to 214.8 cM (7D) and a mean chromosome length of 170.9 cM (Additional file 1: Table S3). SNP density across the three genomes reached 2.2 SNPs/cM. However, this value differed between the subgenomes with 2.5, 3.2 and 0.7 SNPs/cM for the A, B, and D subgenomes, respectively. Also, SNPs were unequally distributed across homeologous chromosomes with the lowest coverage of 613 SNPs (8.1%) on homeologous group 4 and the highest coverage of 1270 SNPs (16.8%) on homeologous group 5 (Additional file 1: Table S3). The highest number of SNPs was mapped to chromosome 5B with 646 SNPs and the lowest number of SNPs to chromosome 4D with 47 SNPs. Over all, the marker distribution showed a high degree of SNP clustering surrounding the centromeres, several major gaps between adjacent SNPs and a low marker density on the D genome. The biggest gap between flanking SNPs was observed on chromosome 7D with 61.5 cM. The same observation was previously reported [2], indicating the low amount of diversity present in the D genome of hexaploid wheat. This finding once again emphasizes the need to selectively increase the number of potentially informative SNPs on arrays originating from the D genome [46].

### LD in WM-800

The analysis of linkage disequilibrium revealed a strong decay of LD with r^2^ = 0.2 at 6.8 cM. The critical value of the 95th percentile of unlinked SNPs was estimated with r^2^ = 0.02 (13.8 cM) (Additional file 2: Fig. S1). The investigation of LD decay per subgenome and per chromosome confirmed the detected pattern, although r^2^ values varied between subgenomes and chromosomes, conforming the results of Neumann et al. [47] and Würschum et al. [48] (Additional file 1: Table S4). The vertical bands of high r^2^ probably arise from incorrect mapping of SNPs to the consensus map (Additional file 2: Figure S1). Similar results were detected for LD on chromosome 5A and 7A [2]. In future, we expect that these questionable mappings may be resolved based on the anticipated completion of the wheat genome sequence. The rapid LD decay within WM-800 corresponded to results from wheat association panels with a mean LD of 3 cM [49]. Compared to already existing MAGIC wheat populations showing a r^2^ = 0.2 within 40 cM in the Australian four-way-MAGIC Wheat population [11], the LD decay of 6.8 cM in WM-800 is quite rapid. We assume that this finding may be attributed to the broader genetic variation, which is present between the founders of WM-800, and to the extra round of intercrossing to combine eight founders rather than four. Both aspects may have resulted in a faster LD decay, which in turn builds an optimal foundation for high-resolution mapping and genome wide association studies. In general, independent from different mapping populations, LD has a slower decay within the D genome compared to the A and B genomes [2, 50], a finding we could confirm in WM-800 (Additional file 1: Table S4).

### Genetic similarity in WM-800

**Fig. 2:**
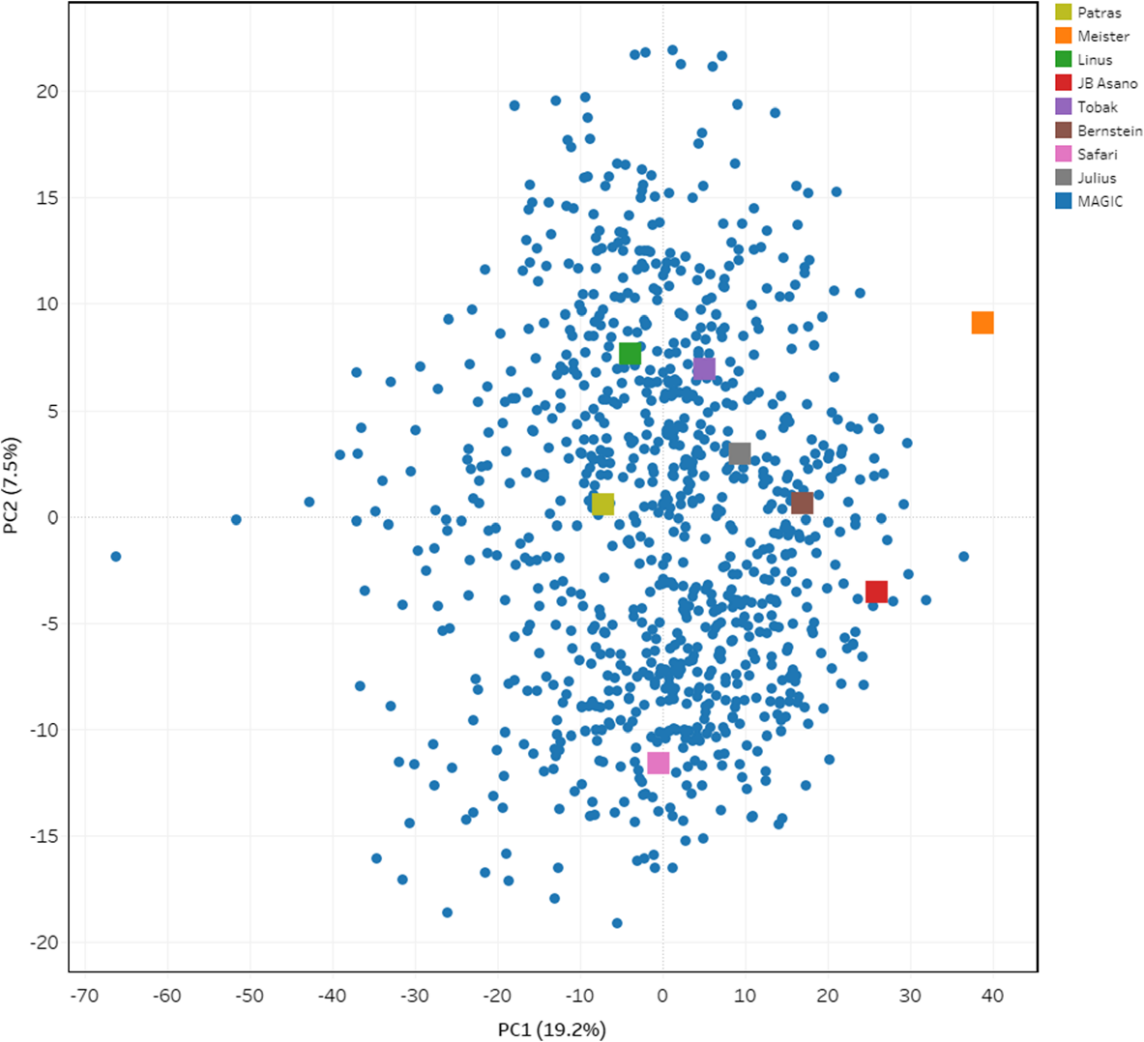
PCA with 910 MAGIC lines and eight founders based on GS estimated from 7849 SNPs

The analysis of genetic similarity (GS) in WM-800 was based on 7849 SNPs and revealed a low degree of genetic similarity among the eight founders, ranging from 51.6% (between Patras and Safari) to 65.5% (between Meister and Bernstein) with a mean GS of 58.1%. These results indicate the high level of diversity between the selected elite soft winter wheat cultivars originating from diverse European breeding programs. Genetic similarity between WM lines ranged from 44.7 to 97.2% with a mean of 59.7% (Additional file 1: Table S5). The analysis of principal components (PCA) was used to investigate structure within the population. The first and the second principle components explained 19.2 and 7.5%, respectively (Fig. 2., Additional file 1: Table S6). Based on the PCA, the WM lines are evenly distributed including the eight founders. We presume that no robust genetic structure is present in WM-800.

### SNP allele frequencies in WM-800

**Fig. 3:**
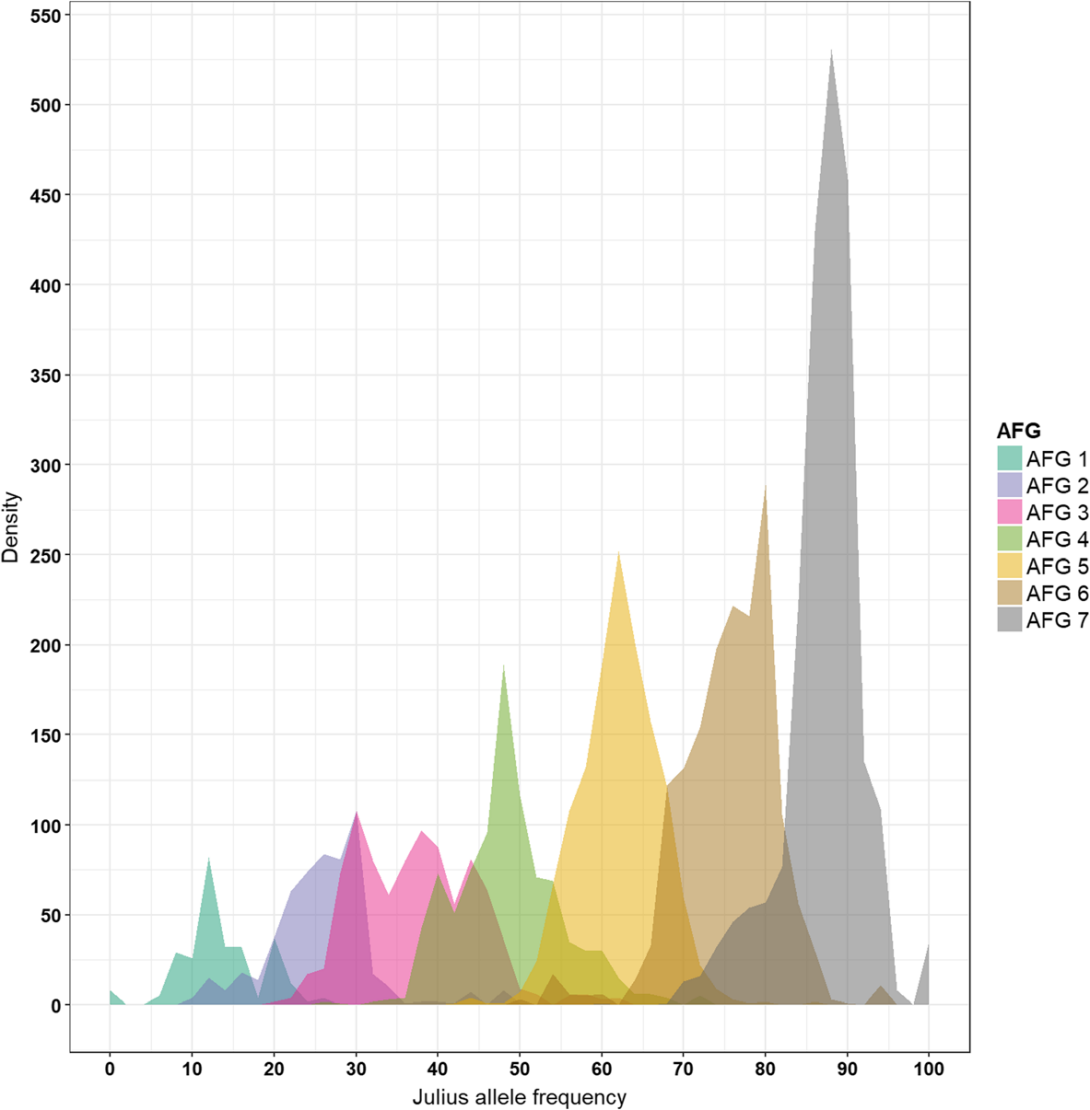
Frequency distribution of Julius alleles per allele frequency group (AFG) 1 to 7

The calculation of the expected SNP allele frequencies in population WM-800 is more complex compared to a bi-parental population. The expected allele frequency in WM-800 depends on the number of founders sharing the same allele. We defined seven allele frequency groups (AFG) depending on the expected allele frequency of the Julius allele (Table 2). Julius is currently used as one cultivar for re-sequencing the hexaploid wheat genome. Therefore, the Julius alleles may be known ahead of other WM founder alleles, being useful for functional annotation of QTL candidate genes in WM-800. In WM-800 the number of SNPs per AFG followed the number of founders sharing the Julius allele, starting with 260 mapped SNPs in AFG 1 and finishing with 2177 mapped SNPs in AFG 7 (Table 2). The mean allele frequency of the Julius allele increased from 13.5 to 87.1% and generally corresponded very well to the expected allele frequency in each AFG. The SNP distribution of the Julius allele frequency shifted towards the right due to the increasing number of SNPs from AFG 1 to AFG 7 (Fig. 3). In total, 2437 mapped SNPs (32.1%) clustered into AFG 1 and AFG 7, indicating the presence of unique SNP alleles in WM-800, which can be traced back to a single WM-800 founder, either Julius (in AFG 1) or one of seven Non-Julius founders (in AFG 7) (Table 2). We consider the frequency of 32.1% unique SNPs in WM-800 as very high. This observation may support the power of a subsequent QTL detection since no additional founders will dilute the true effect of a founder QTL allele. Among the mapped SNPs, the maximum and minimum number of unique SNPs originated from Safari (5.3%) and Meister (2.6%) (Table 2). This finding indicates a relatively even distribution of unique SNPs among the eight WM founders and supports the results of the genetic diversity study.

**Table 2:** SNP segregation in seven allele frequency groups (AFG) and expected, observed frequency of Julius alleles

Allele frequency group	No of founders carrying the Julius allele	Unique SNPs (%)	Observed Julius allele frequency (%)	Expected Julius allele frequency (%)	Unique SNPs (%)
AFG 1 - Unique Julius	1	260	3.4	13.5	12.5
AFG 2	2	531		25.9	25.0
AFG 3	3	877		37.2	37.5
AFG 4	4	842		48.5	50.0
AFG 5	5	1315		62.2	62.5
AFG 6	6	1582		75.7	75.0
AFG 7	7	2177		87.1	87.5
AFG 7 subgroups					
AFG 7 - Unique Patras		368	4.9		
AFG 7 - Unique Meister		200	2.6		
AFG 7 - Unique Linus		382	5.0		
AFG 7 - Unique JB Asano		233	3.1		
AFG 7 - Unique Tobak		265	3.5		
AFG 7 - Unique Bernstein		329	4.3		
AFG 7 - Unique Safari		400	5.3		
Sum of unique SNPs = AFG 1 + AFG7		2437	32.1		
Total number of SNPs		7584

### Segregation distortion in WM-800

Segregation distortion (SD) is a common phenomenon in plant genome analysis and has been described in many species, like barley [51, 52], triticale [53] and wheat [13, 40, 54]. It is the deviation of the segregation ratio of a locus from the expected Mendelian ratio [55]. The allele frequency depends on the crossing scheme. In an eight-way MAGIC population it follows a 1:1:1:1:1:1:1:1 segregation, provided that all eight founder alleles can be differentiated. In case of genotyping bi-allelic SNP markers in WM-800, the segregation is reduced to two classes, in our case: Julius allele and Non-Julius allele. Here, the expected allele frequencies depend on the number of founders sharing the Julius allele as indicated in Table 2. Genetic causes for deviation from the expected segregation are selection, either natural or artificial. Allelic selection may take place during gametogenesis or during embryogenesis. However Dreissig et al. [52] and Belanger et al. [56] reported that gametic selection was almost absent in contrast to zygotic selection, when they compared SNP genotyping in pollen and doubled haploids of barley. Zygotic selection may occur during population development and propagation where fitness disparities between alleles or genotypes may result in reduced reproduction rates or even lethality. The latter case is also termed hybrid necrosis caused by genes involved in immunity reactions [57–59]. In all cases, the frequency of the allele or genotype under negative selection is reduced in the final population. In addition, also those alleles, which are genetically linked and, in most cases, jointly inherited to the next generation, will be reduced in the final population depending on the genetic distance between the linked allele and the causative gene under selection.

**Fig. 4:**
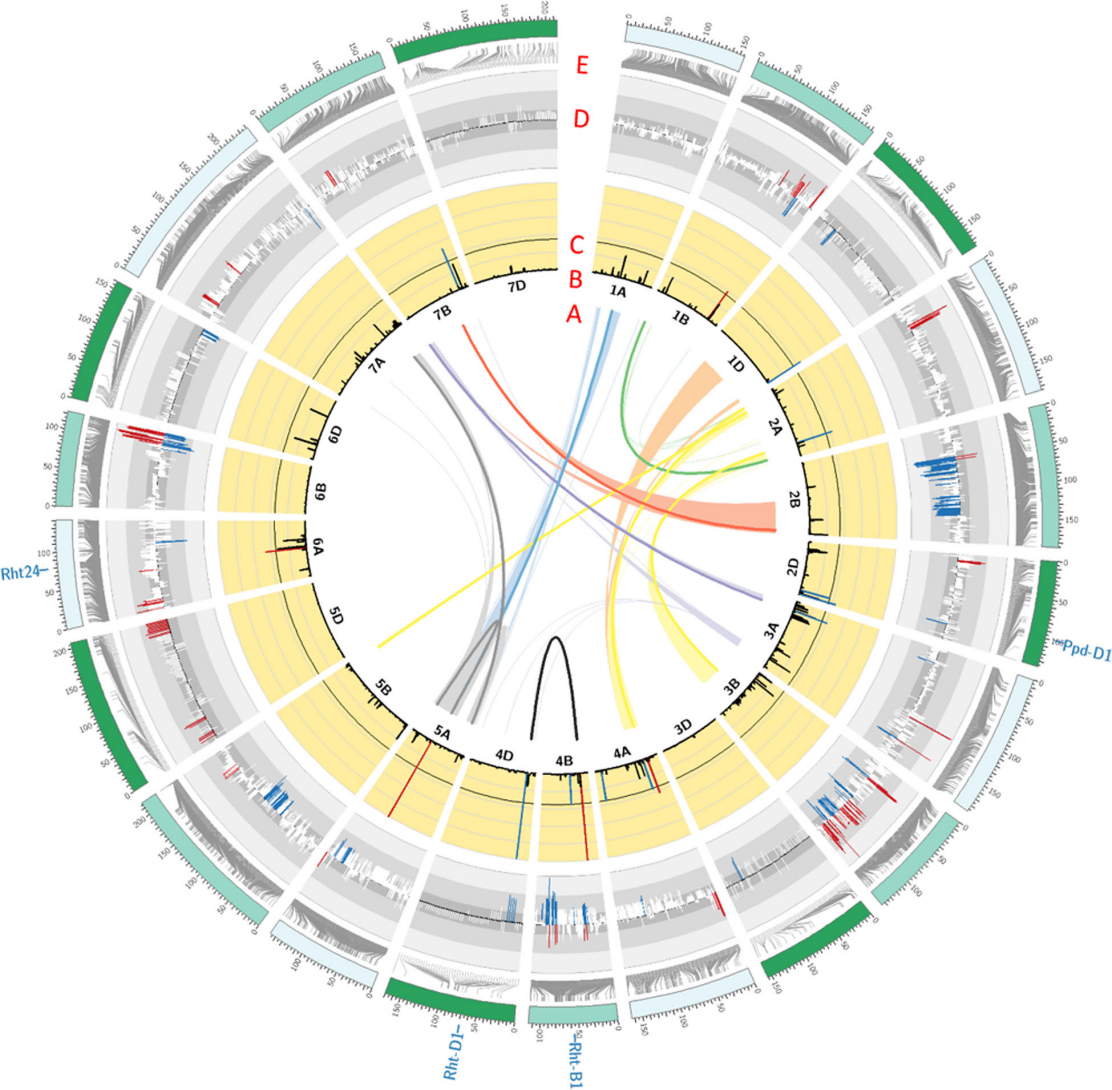
Circos plot illustrating QTL controlling plant height and SNPs exposed to segregation distortion in WM-800. **a**) Links in the circle represent significant (P_FDR_ ≤ 0.01) di-genic epistatic interactions between independent SNPs. The firm lines connect the epistatically interacting SNPs remaining after forward and backward selection. **b**) Chromosome 1A to 7D. **c**) Bars represent the detection rate of SNPs in 100 cross-validation runs during GWAS. The dark grey line represents the detection rate threshold of ≥35%. Coloured bars indicate the 14 significant SNPs identified to control plant height. Red and blue bars symbolize enhancing and reducing effects of the homozygous Julius allele on plant height in WM-800, respectively. **d**) Segregation distortion regions along the chromosomes. Height of bars symbolized the strength of deviation of the expected Julius allele frequency. Blue and red colours indicate SNPs revealing a decrease and an increase of the Julius allele frequency, respectively. E) Grey connector lines represent the genetic position of the SNP on the wheat chromosome. Position of candidate genes *Rht-D1*, *Rht-B1, Rht24* and *Ppd-D1* are indicated outside the Circos plot

Segregation distortion in WM-800 was investigated by chi square testing of each SNP according to the expected allele frequency in each AFG (Table 2). To our knowledge, this AFG-specific approach of the investigation of segregation distortion is applied for the first time to a plant MAGIC population. In WM-800, 1417 mapped out of 7584 (18.7%) SNPs revealed significant SD with p_Bon-Holm_ ≤ 0.01 (Additional file 1: Table S7). Out of these, 877 SNPs were placed in SDR. SDRs were not evenly distributed across the wheat subgenomes: 210, 612 and 55 distorted SNPs were mapped to the A, B and D subgenomes, respectively. These numbers translate into 6.7, 16.7 and 6.8% of the SNPs of subgenomes A, B and D, respectively, which were located in distorted segregation regions. SNPs located on subgenome B, thus, were much more frequently exposed to selection, either natural or artificial, during the development of WM-800, than SNPs on subgenomes A and D. Distorted SNPs were predominantly found on chromosomes 2B, 3B, 4B, 5A, 5B, 5D and 6B, where between 14.1 and 32.1% of all chromosomal SNPs showed distorted segregation (Fig. 4, Additional file 1: Table S7).

The proximal segregation distortion region in wheat on chromosome 2B was already reported [40, 54, 60–63]. Since the onset of modern wheat breeding, alien chromosome fragments have been incorporated into bread wheat germplasm to broaden the genetic base, for instance, to improve pathogen resistance. For example, the stem rust and powdery mildew resistance genes *Sr36* and *Pm6* were introgressed from the short arm of chromosome 2G of *T. timopheevii* [13], which, presumably, resulted in segregation distortion [54, 64]. In WM-800, the founders Meister and JB Asano also contributed the alien *Pm6* gene*.* However, the majority of distorted SNPs were in favour of the Tobak allele suggesting that not the alien fragment carrying founders Meister and JB Asano alone are responsible for SDR on chromosome 2B in WM-800. In future, we propose to build haplotype genotypes based on selective exome capture sequencing of the wheat gene space, in order to potentially differentiate all eight founder alleles [65, 66]. This way, the identification of individual alleles, positively or negatively selected in WM-800, may be enhanced. Furthermore, chromosome 2B was reported to host genetic regions responsible for plant regeneration [53]. Potentially genes linked to *Ppd* might be involved within the process [67]. Also, studies in rice and maize reported SDRs located close to genetic regions harbouring gametophytic factors [68, 69].

In contrast to Gardner et al. [13], segregation distortion on chromosome 1B was relatively moderate in WM-800. Chromosome 1B harbours the rye translocation 1RS:1BL, which is known to induce SD [70, 71]. However, the diagnostic SNP TG0025 for 1RS:1BL was monomorphic between all founders of WM-800. Therefore, we assume that segregation distortion for chromosome 1B did not arise through the translocation 1RS:1BL as discovered in several publications [13, 72, 73]. Chromosome 3B harboured the highest percentage (32.1%) of SNPs in SDR in WM-800. Population specific segregation distortion on chromosome 3B was also observed in the MAGIC NIAB2015 population [13] and to a lower degree in two wheat RIL populations [73].

The only evidence for artificial selection in WM-800 was found on chromosome 4D**.** Four SNPs, clustering into one SDR, showed a significant deviation from the expected Mendelian segregation (Additional file 1: Table S7 and S1). This SDR included the SNPs TG0011a and TG0011b, which are diagnostic for the plant height reducing gene *Rht-D1*. The distorted SNPs were skewed against the semi-dwarf allele *Rht-D1b* with a 13.79% lower allele frequency than expected. In WM-800, *Rht-D1b* was simultaneously inherited by four founders, Julius, Patras, Linus and JB Asano. During development of WM-800, 170 double dwarfs, simultaneously containing semi-dwarf alleles at *Rht-D1b* and *Rht-D1b,* were removed in generation F_4_ from 1323 WM-lines based on two diagnostic KASP markers in order to avoid the presence of low-yielding WM lines in the following field studies. Thus, the artificial selection against double dwarfs resulted in a significantly reduced presence of semi-dwarf alleles at the *Rht-D1 locus.* However, the frequency of the second semi-dwarf allele, *Rht-B1b* on chromosome 4B*,* was only slightly, but non-significantly, reduced by 3.08%, although this allele was inherited by two WM-800 founders, Tobak and Safari.

In general, a large portion of SNPs in SDRs belong to allele frequency groups AFG 1 and 7, i.e. 49 and 280 SNPs (Additional file 1: Table S7). Investigating SDR in AFG 1 and 7 is helpful to identify chromosomes, which are selected in favour or against a particular WM founder. In total, ten major selection events of single founders were identified in WM-800. More than 10 independent SNPs are present, showing an increase of unique alleles from WM founders Tobak (2B), Safari (3B), Linus (4B), Safari (5A), Julius (5B) and Bernstein (6B). Likewise, more than 10 independent SNPs are present, showing a significant decrease of unique alleles from WM founders Patras and Linus (3B), Julius (5B) and Meister (6B) (Additional file 1: Tables S7 and S8). Only WM founder JB Asano was not involved in any major selection event. This finding may indicate the existence of adaptive selection between founder alleles during population development where individual chromosomal segments were positively or negatively selected. It is noticeable that most chromosomes involved in major selection events belong to subgenome B. This finding is in agreement with Gardner et al. [13], who also identified a preponderance of B chromosomes showing SD.

### Genome-wide association studies for plant height

The relatively low average genetic similarity of 59.7% between WM-800 lines, the strong phenotypic variation of plant height and its high heritability are promising features for a successful GWAS. Consequently, 14 highly significant QTL were estimated regulating plant height in WM-800 with p_Bon-Holm_ ≤ 0.001 (Table 3, Fig. 4). In total, these QTL explained 59.9% of the cumulated genotypic variance (R^2^val). Only four QTL explained more than 4% of the genetic variation. This indicates, that plant height in WM-800 is controlled by a small number of genes revealing strong effects and a larger number of genes exposing small effects. However, approximately 50% of the genetic variation remains unexplained. This hidden part of genetic control may point to an even larger number of additional genes with small effects. To locate these QTL, an increase of population size, marker density and number of tested environments may be necessary.

**Table 3:** List of 14 QTL controlling plant height in wheat population WM-800 (detection rate ≥ 35)

QTL name^a^	SNP marker^b^	Chr^c^	Pos^d^	Range^e^	DR^f^	P_Bon-Holm_^g^	Effect^h^	R^2^val^i^ (%)	Candidate gene^j^	Literature^j^
QHEI.WM-800.1B	CAP8_c5043_190	1B	117.8	117.8	39	6.70E-05	3.8	0.8		
QHEI.WM-800.1D	BS00063511_51	1D	167.1	167.1	44	9.24E-05	−3.9	1.4		[16]
QHEI.WM-800.2A	Excalibur_c20439_825	2A	148.8	148.8	42	6.69E-05	− 2.4	1.0		
QHEI.WM-800.2D	Excalibur_rep_c67599_2154	2D	97.1	97.1	35	9.37E-06	−3.5	1.3	*Ppd-D1*	[89]
QHEI.WM-800.3A	IAAV5729	3A	61.1	61.1	41	5.01E-05	−2.9	1.3		[26]
QHEI.WM-800.4A.a	wsnp_Ex_c5487_9686018	4A	43.2	43.2	42	7.25E-05	2.8	0.8		
QHEI.WM-800.4A.b	Kukri_c48199_102	4A	49.0	49.0	36	4.76E-05	− 2.8	1.0		
QHEI.WM-800.4A.c	wsnp_Ex_c12725_2021270	4A	147.2	147.2	35	4.69E-05	−4.5	0.5		
QHEI.WM-800.4B.a	TG0010a	4B	56.0	56.0	100	1.25E-47	12.8	6.7	*Rht-B1*	[74]
QHEI.WM-800.4B.b	BS00030843_51	4B	62.9	62.9	35	3.97E-05	−2.8	0.6		
QHEI.WM-800.4D	TG0011a	4D	69.2	69.2	99	4.17E-20	−14.9	21.5	*Rht-D1*	[74]
QHEI.WM-800.5A	RAC875_c30711_544	5A	67.0	67.0	100	1.38E-07	5.3	5.5		
QHEI.WM-800.6A	tplb0047k12_1370	6A	79.1	79.1 (2)	47	1.44E-16	5.1	4.8	*Rht24*	[76]
QHEI.WM-800.7B	RAC875_c76528_296	7B	155.4	155.4–159.7 (2)	51	2.45E-05	−4.3	0.8		
								59.9		

^a^QTL name including trait, population and chromosome information

^b^SNP marker name [36]

^c^Chromosome of SNP marker [36]

^d^Genetic position of SNP marker [36]

^e^Range of QTL in cM including number of significant SNPs (in brackets) within the range

^f^Detection rate (DR) of SNP by cross validation

^g^Bonferroni corrected *p*-value of SNP marker

^h^Effect of homozygous Julius allele (in cm) compared to non-Julius allele

^i^Cross-validated proportion of explained genetic variance of validation set

^j^Candidate genes with references

The strongest QTL controlling plant height in WM-800 are the semi-dwarf genes *Rht-D1* and *Rht-B1* on chromosomes 4D and 4B [74]. They explain 21.5% (*Rht-D1*) and 6.7% (*Rht-B1*) of the genotypic variance (Table 3, Additional file 1: Table S1), This findings are in correspondence with results in association panel [16] and in the Australian four-parent MAGIC Wheat population [11]. The gibberellic acid (GA) insensitive semi-dwarfing alleles *Rht-B1b* and *Rht-D1b* are presumably the most studied plant height reducing genes in wheat. Together, they are present in probably 90% of the world’s semi-dwarf wheat cultivars, strongly reinforcing green revolution in wheat [75]. The homozygous Julius alleles at these two QTL increased plant height relative to the Non-Julius alleles by 12.8 cm (*Rht-B1*) and reduced plant height by − 14.9 cm (*Rht-D1*), respectively. These effects are in accordance with the expected effects inherited by the WM founders (Fig. 1). The Non-Julius founders Tobak and Safari possess the semi-dwarf allele *Rht-B1b* whereas the founders Julius, Patras, Linus and JB Asano possess the semi-dwarf allele *Rht-D1b* (Table 1). Locating both semi-dwarf genes, *Rht-B1* and *Rht-D1*, with high precision within the MAGIC WM-800 population may serve as a proof of concept. This finding may support the establishment of WM-800 as a precious genetic resource to be used in high resolution mapping and, ultimately, cloning of quantitative genes controlling developmental and agronomic traits in the elite wheat gene pool.

A further strong plant height QTL in WM-800 was located on chromosomes 6A, 79.1 cM, explaining 4.8% of the genotypic variation. At this locus the Julius allele resulted in a 5.1 cm increase in plant height. The QTL QHEI.WM-800.6A coincided with results from an association panel [76] and from a bi-parental population [77]. The effect can be attributed to the *Rht* locus *Rht24*.

In addition to the strong effects mentioned before, a QTL in close proximity to the photoperiod sensitivity gene *Ppd-D1* was detected on chromosome 2D. Here, the Julius allele was associated with a plant height reducing effect of − 3.5 cm. The *Ppd-D1* effect on plant height was also reported in wheat [11, 16, 78] and in barley [79], indicating that *Ppd-D1* may be involved in controlling a number of developmental traits.

So far, no further candidate genes can be associated to the remaining ten significant QTL regions controlling plant height. Among the numerous small effects, the QTL region on chromosome 5A with an effect of 5.3 cm and 5.5% of explained genotypic variation harbours great potential for plant height reduction. In future, a follow up study based on exome capture sequencing of informative, recombinant WM offspring lines may be used to fine-map the QTL region and ultimately identify the causative gene. For this, a pair of WM offspring lines need to be selected that segregate for the SNP under investigation and, simultaneously, is fixed for the long straw allele at the remaining 13 plant height QTL regions. This pair can be developed from a WM line heterozygous at the QTL under investigation, following the heterogeneous inbred family (HIF) concept proposed by Tuinstra et al. [80] and successfully applied, for instance, by Liu et al. [81].

### Epistatic effects on plant height

Epistasis refers to a genetic interaction between two or more loci in a genome [82]. Epistasis was used to dissect the genetic architecture of complex traits like flowering time in crops [83]. In WM-800, the analysis of epistatic interactions was based on a di-genic model. In total, eleven epistatic marker*marker interactions controlling plant height were located (Fig. 4). Altogether, these interactions explained 84.1% of the epistatic variance with R^2^ values ranging from 3.4 to 45.1% (Table 4). In contrast, the estimated epistatic interactions explained only between 0.0 and 3.0% of the genetic variance for plant height in a set of European winter wheat cultivars [16]. However, a high level of explained genetic variance (77%) for flowering time was observed in a NAM barley population [4]. The latter authors emphasized (1) the use of multi-parental populations to investigate complex traits, (2) the necessity to investigate epistatic interactions in order to estimate the “missing heritability” and (3) the discovery of, so far, unknown functional gene networks by modelling epistatic effects.

**Table 4:** Significant epistatic interaction effects for plant height in WM-800

Epistatic Interaction^a^	SNP M1^b^	Chr^c^	Pos^d^	SNP M2^b^	Chr^c^	Pos^d^	FDR^e^	R^2f^	J/J^g^	N/N^h^	J/N^i^	N/J^j^	aa^k^
EpiHEI.WM-800.1	Kukri_c67601_267	1A	71.5	**TA001269–1282**	**5A**	**62.7**	3.23E-13	17.0	81.4	83.6	90.4	75.3	0.70
EpiHEI.WM-800.2	BS00050522_51	1B	5.3	**Kukri_c21008_657**	**2A**	**151.3**	2.35E-03	6.4	80.6	81.8	81.8	72.2	8.40
EpiHEI.WM-800.3	**Excalibur_c92298_213**	**1D**	**171.3**	wsnp_Ex_c539_1072859	4A	60.4	1.24E-02	5.4	81.3	81.1	78.1	82.3	1.80
EpiHEI.WM-800.4	BobWhite_c11022_78	2A	11.5	**TA004056–0809**	**4A**	48.5	1.38E-02	3.4	80.3	80.6	87.4	79.9	−6.40
EpiHEI.WM-800.5	RAC875_c47161_100	2A	47.2	TA004556–0473	5B	176.6	3.44E-04	8.6	82.0	78.6	81.8	80.1	−1.40
EpiHEI.WM-800.6	**RAC875_c9523_328**	**2A**	**144.2**	BS00095061_51	3B	67.5	1.35E-02	4.5	81.5	81.1	78.0	81.9	2.60
EpiHEI.WM-800.7	TA002989–0535	2B	157.2	RAC875_rep_c78007_425	7B	135.4	5.95E-09	9.8	78.4	96.2	81.3	79.6	13.70
EpiHEI.WM-800.8	**BS00067499_51**	**3A**	**68.7**	Tdurum_contig57370_82	7B	53.8	4.67E-04	9.7	79.8	77.4	81.9	86.7	−11.40
EpiHEI.WM-800.9	**TG0010a**	**4B**	**56.0**	**TG0011a**	**4D**	69.2	6.32E-44	45.1	73.9	77.7	89.0	NA	−13.20
EpiHEI.WM-800.10	wsnp_Ra_rep_c69221_665	5A	42.0	Tdurum_contig17062_221	7A	202.2	3.58E-06	11.5	82.4	77.0	90.0	78.4	−9.00
EpiHEI.WM-800.11	**TA001269–1282**	**5A**	**62.7**	wsnp_Ex_c1880_3545329	5A	104.9	3.49E-11	15.2	76.9	82.8	82.4	88.7	−11.50
								84.1					

^a^Name of epistatic interaction including prefix Epi, trait, population and a consecutive number

^b^Interacting SNP markers M1 and M2

^c^Chromosomal location of SNP [36]

^d^Genetic position in cM of SNP [36]

^e^FDR corrected *p* value of marker 1*marker 2 interaction

^f^Proportion of explained epistatic variance in % of marker 1*marker 2 interaction (in %)

^g^Mean plant height (in cm) of WM lines carrying homozygous Julius alleles (J) at both loci

^h^Mean plant height (in cm) of WM lines carrying homozygous Non-Julius alleles (N) at both loci

^i^Mean plant height (in cm) of WM lines carrying a homozygous Julius allele (J) and a Non-Julius allele (N) at loci 1 and 2, respectively

^j^Mean plant height (in cm) of WM lines carrying a homozygous Non-Julius allele (N) and a Julius allele (J) at loci 1 and 2, respectively

^k^Estimated additive by additive epistatic interaction effect (aa) [45]

Bold letters indicate markers located in regions containing main QTL for plant height (Table 3)

NA = data not available, in this case, the epistatic interaction effect was estimated by adding twice the mean plant height of the JN group

In WM-800, the semi-dwarf genes *Rht-B1* and *Rht-D1,* located on chromosomes 4B and 4D, proved to be the major players of di-genic epistatic interactions controlling plant height. This interaction explained a maximum of 45.1% of the epistatic variance and gave rise to an estimated additive by additive epistatic effect (aa) of − 13.2 cm (Table 4). WM lines possessing two Julius or two Non-Julius alleles at *Rht-B1* and *Rht-D1* revealed a mean plant height reduction by − 13.2 cm compared to WM lines possessing a Julius and a Non-Julius allele at both loci. This finding is in agreement with studies of Ellis et al. [74] and Baenziger et al. [84]. More recently, it was reported that wheat genotypes possessing both semi-dwarf alleles simultaneously produced significantly shorter plants [11]. In contrast, several studies on doubled haploid (DH) and recombination inbred line (RIL) populations did not detect the major plant height interaction effect between *Rht-B1b* and *Rht-D1b* [85–87], possibly due to the presence of low genetic diversity between the parents. The same holds true for Würschum et al. [16] and Zhao et al. [86] who studied epistatic interactions in panels of wheat cultivars and hybrids, respectively. Finding the *Rht-B1b* by *Rht-D1b* epistatic interaction in WM-800 may serve as a proof of concept to include epistatic models in MAGIC populations.

**Fig. 5:**
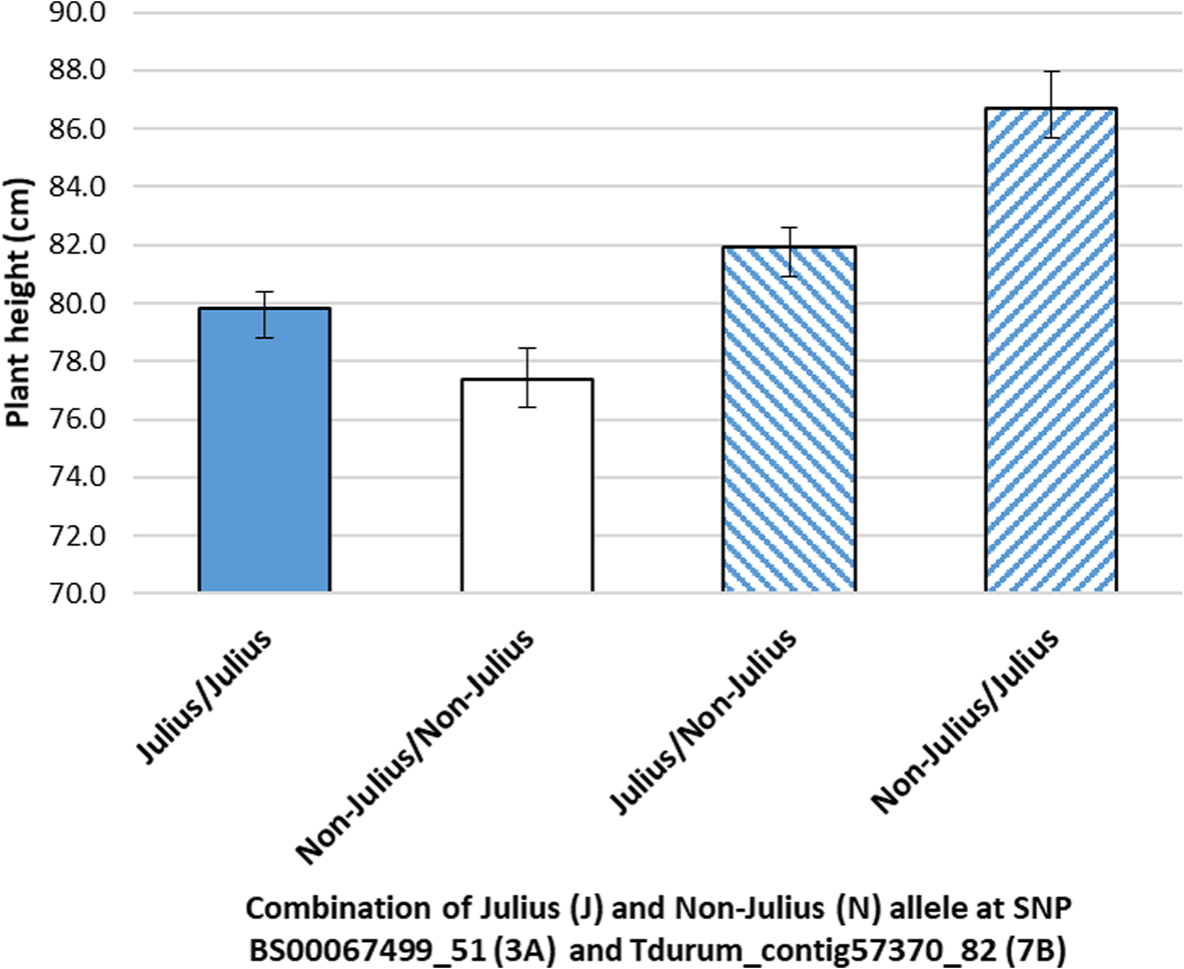
Epistatic effect of EHEI.WM-800.8. at chromosome 3A, 68.7 cM and 7B, 53.8 cM

In addition to the *Rht-B1* by *Rht-D1* interaction ten further epistatic interactions were detected for plant height in WM-800 (Table 4). In most cases (8 out of 11) at least one marker involved in epistasis was associated with a QTL main effect. This finding is in accordance with others who also found markers, which were simultaneously associated with main QTL effects and epistatic effects in wheat [86] and maize [88], respectively. Additional strong epistatic effects were found between markers on different chromosomes, for example between SNPs on chromosomes 3A and 7B with an epistatic effect of − 11.4 cm (Fig. 5). Epistatic interactions were also found between independent SNPs on the same chromosome, for instance on chromosome 5A producing an estimated additive by additive epistatic effect of − 11.5 cm (Table 4). So far, no candidate genes are available to explain these epistatic interactions. Our findings support the idea that epistatic interactions need to be taken into account to explain a maximum of the genetic variation present in MAGIC populations. WM-800 serves as an ideal source to apply epistatic models. The population is larger than standard mapping populations. The chance to detect epistatic interactions is increased because eight rather than two alleles segregate at each investigated locus. In future, we propose to increase the number of genotyped markers in WM-800 and to transform bi-allelic SNP marker into multi-allelic haplotype markers in order to increase the odds to detect epistatic interacting loci and to fine-map and, ultimately, clone the causative genes, which epistatically interact in a gene network.

## Conclusion

The genetic structure of the multi-parental population WM-800 enables to conducted detailed studies on the genetic architecture of important agronomic traits in wheat, exemplified by plant height as a proof of concept. In the present study we demonstrated that plant height in WM-800 is mainly determined by large-effect QTL and di-genic epistatic interactions. The semi-dwarf genes *Rht-B1 and Rht-D1* turned out to be prominent in both cases. In addition, a number of regions, predominantly on the subgenome B chromosomes 2B, 3B, 4B, 5B and 6B, could be identified to suffer from segregation distortion. Most SDRs turned out to be WM founder specific, indicating a predominantly subgenome B chromosome-specific selection against or in favour of WM founder alleles during the development of WM-800. Although no artificial selection was applied except for the occurrence of double dwarfs on chromosomes 4B and 4D (*Rht-B1 and Rht-D1).*

Our first findings demonstrate the high value of WM-800 to support both - genetic studies to explain genetic networks regulating quantitative traits as well as breeding improved wheat cultivars. Regarding the latter aspect, we propose to evaluate WM-800 for direct selection of improved winter wheat cultivars. Both routes are currently followed up in our MAGIC-WHEAT consortium consisting of wheat geneticists and applied wheat breeders.

## Additional files

Additional file 1 **Table S1.** SNP genotypes, plant height, QTL detection rate and genotype and allele frequencies for 910 MAGIC WHEAT lines and eight founders. **Table S2.** Plant height (cm) raw data for MAGIC WM-lines and founders for year 2015 and 2016. **Table S3.** Distribution of polymorphic SNPs within WM-800 according to genome positions of Wang et al.... [36]. **Table S4.** Mean, minimum, maximum and coefficient of variation (CV, in %) of linkage disequilibrium (r2) in WM-800, calculated per chromosome, subgenome and across the whole wheat genome. **Table S5.** Genetic similarity (GS) between founders and WM-800 lines based on 7849 polymorphic SNPs. **Table S6.** Principle Component Analysis (PCO) based on genetic similarities (GS) between founders and WM-800 lines**. Table S7.** Distribution of segregation distortion (SD) SNPs and segregation distortion regions (SDR) across the wheat genome and across allele frequency groups**. Table S8.** Selection of unique SNPs from AFG 1 and AFG 7 in WM-800. Major selection events, represented by > 10 SNPs, are indicated in bold. (ZIP 2660 kb)

Additional file 2: **Figure S1.** Linkage disequilibrium (r^2^) as a function of genetic distance (mean of whole genome) within WM-800. The horizontal line (0.02) indicates the 95th percentile of the LD distribution of unlinked pairs of loci, representing the population-specific critical r^2^ value. The curve (red line) was fitted with second-degree LOESS. (PNG 30 kb)

## Abbreviations

AFG: Allele frequency group
cM: Centimorgan
F_1_: First generation after initial cross
F_2_: Second generation after initial cross
F_4_: Fourth generation after initial cross
F_4:5_: Fifth generation derived from fourth generation
F_4:6_: Sixth generation derived from fourth generation
GA: Gibberellic acid
GB: Giga base
GS: Genetic similarity
GWAS: Genome wide association study
KASP: Kompetitive allele specific PCR
LD: Linkage disequilibrium
MAGIC: Multi-parent advanced generation intercross
MNI: Mean imputation
NAM: Nested association mapping
PC: Principal component
PCA: Principal component analysis
QTL: Quantitative trait locus/loci
Rht: reduced height
RIL: Recombinant inbred line
SNP: Single nucleotide polymorphism
SSD: Single seed descent
