# Supplementary figures and images for: Adaptive selection of founder segments and epistatic control of plant height in the MAGIC winter wheat population WM-800
Source: BMC Genomics. 2018 Jul 31;19:559. doi: 10.1186/s12864-018-4915-3 (PMC6069784; doi:10.1186/s12864-018-4915-3)

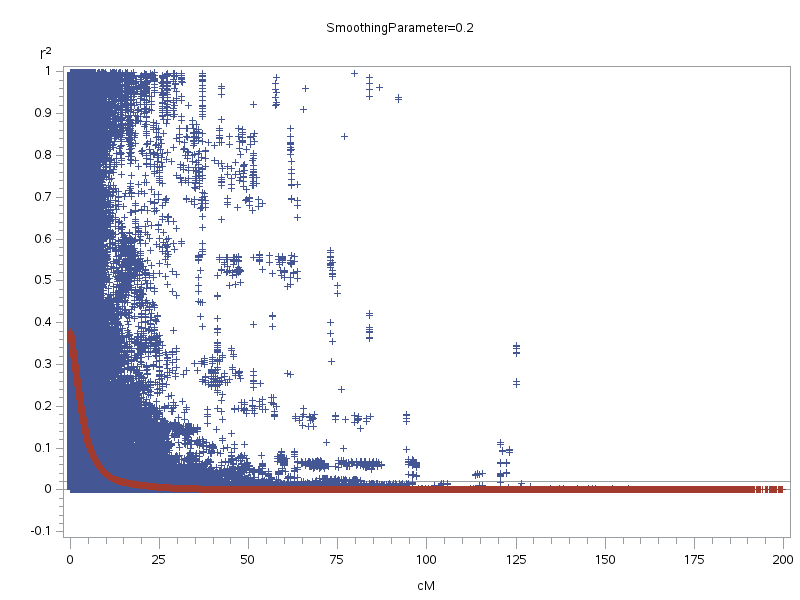

Supplement: Supplementary file 2 — Figure S1. Linkage disequilibrium (r2) as a function of genetic distance (mean of whole genome) within WM-800. The horizontal line (0.02) indicates the 95th percentile of the LD distribution of unlinked pairs of loci, representing the population-specific critical r2 value. The curve (red line) was fitted with second-degree LOESS. (PNG 30 kb) [file 12864_2018_4915_MOESM2_ESM.png]
